# Hepatitis C Infection Is Not a Cardiovascular Risk Factor in Young Adults

**DOI:** 10.3390/biomedicines12102400

**Published:** 2024-10-20

**Authors:** Paweł Rajewski, Małgorzata Pawłowska, Dorota Kozielewicz, Dorota Dybowska, Anita Olczak, Jakub Cieściński

**Affiliations:** 1 Department of Internal and Infectious Diseases, Provincial Infectious Disease Hospital, 85-030 Bydgoszcz, Poland; j.ciescinski@wsoz.pl; 2 Faculty of Health Sciences, University of Health Sciences in Bydgoszcz, 85-067 Bydgoszcz, Poland; 3 Department of Infectious Diseases and Hepatology, Faculty of Medicine, Collegium Medicum Bydgoszcz, Nicolaus Copernicus University, 87-100 Torun, Poland; mpawlowska@cm.umk.pl (M.P.); d.kozielewicz@wsoz.pl (D.K.); d.dybowska@wsoz.pl (D.D.); anita_olczak@interia.pl (A.O.)

**Keywords:** HCV, cardiovascular disease risk factor, young adults, coronary artery disease, cardiovascular disease

## Abstract

**Background**: Cardiovascular diseases are one of the leading causes of hospitalization and death in Poland and around the world and are still an ongoing problem for modern medicine. Despite advances in diagnosis and treatment, both conservative and invasive, the prevention of cardiovascular disease directed at reducing risk factors remains a problem. The main classical risk factors for the development of cardiovascular disease in Poland include hypertension, lipid disorders, obesity, diabetes and smoking. A new non-classical risk factor is HCV infection. Most of the studies on the impact of HCV infection on cardiovascular disease involve elderly populations with long-term infections and advanced liver fibrosis. **Methods**: Hence, we set out to analyze the prevalence of risk factors and cardiovascular disease in a population of young adults under 45 years of age infected with HCV, according to gender, HCV genotype and the duration of infection. The study group consisted of 217 patients of both sexes aged 21 to 45 years (mean age 36 years). **Results**: No cardiovascular disease was found among the young adults infected with HCV in the study group. The most common risk factor was cigarette smoking, which affected 20.7% of the subjects, followed by hypertension (12%) and diabetes mellitus (5.5%); the prevalence was lower than in the general population. Most of the patients were characterized as overweight, with a mean BMI of 26.39 kg/m^2^. The mean values of other metabolic parameters—total cholesterol, LDL, HDL, uric acid and glucose—were within the population norm. The mean value of CRP was 1.43, which may indicate a moderate cardiovascular risk. **Conclusions**: Based on the conducted research, it was found that HCV infection in young individuals was not a risk factor for cardiovascular diseases, and the prevalence of risk factors was similar to that in the general population. The effect of HCV on the increase in C-reactive protein requires further study. The early detection of HCV infection and treatment can be considered as a prevention of cardiovascular disease.

## 1. Introduction

Atherosclerosis-related cardiovascular disease (ASCVD, atherosclerotic cardiovascular disease), which includes ischemic heart disease, ischemic stroke or peripheral vascular disease, is the most common and important cause of morbidity, hospitalization and mortality in Poland and worldwide [1,2,3,4,5,6,7,8,9,10].

The main risk factors for the development of cardiovascular disease in Poland include hypertension, lipid disorders, obesity, diabetes and smoking (Table 1) [6,7,8,9,10].

Chronic hepatitis C is one of the leading causes of chronic liver disease worldwide and one of the most common causes of cirrhosis and hepatocellular carcinoma, contributing to a reduced quality of life and life expectancy.

It is estimated that there are 71 million patients with chronic hepatitis C worldwide (0.5–2.3% of the population). In Poland the percentage of people with positive anti-HCV antibodies is about 1% of the population and with confirmed infection is 0.6%; however, these figures may be underestimated, due to the lack of screening. The risk of HCV is due to years of sparse or asymptomatic courses, low detection rates and lack of immunizations [11,12,13,14,15,16].

Studies in recent years show that HCV infection also leads to the development of metabolic disorders, which play an important role as a risk factor for the development of cardiovascular disease [17,18,19,20,21,22].

The noticeable effect of HCV on the development of obesity, insulin resistance, the development of pre-diabetes, diabetes, lipid disorders or hepatic steatosis has led to the metabolic disorders in the course of HCV infection being called “metabolic-viral syndrome” by some authors and hepatic steatosis being described is as an organ form of metabolic syndrome [19,20,21,22].

The observed increased incidence of cardiovascular disease in patients with hepatitis C has also contributed to HCV being considered in recent years as a new, non-classical risk factor for cardiovascular disease and the complications that occur in this regard as an extrahepatic manifestation of HCV infection. The potential role of HCV as a risk factor for the development of cardiovascular disease is complex. On the one hand, the infection directly leads to chronic inflammation, contributing to the development of atherosclerosis and vascular endothelial dysfunction; on the other hand, it leads to the development of the aforementioned metabolic disorders, which have been recognized as classic cardiovascular risk factors for years [17,18,19,20,21,22,23,24,25,26].

Most of the studies evaluating the impact of HCV infection on the development of metabolic disorders and cardiovascular disease involve adult and elderly populations with long-standing chronic hepatitis C, so we decided to examine the impact of HCV infection on the development of these conditions in a population of young adults under 45 years of age [19,22].

### Purpose of the Work

The aim of this study was to analyze the prevalence of selected cardiovascular risk factors and the incidence of cardiovascular disease among young adults up to 45 years of age with chronic hepatitis C and to assess their association in relation to gender, age, the duration of infection, progression of liver fibrosis and HCV genotype.

## 2. Material and Methods

The study group consisted of 217 patients, 96 women and 121 men (58% male and 42% female) aged between 21 and 45 years (mean age 36 years) diagnosed with chronic hepatitis C qualified for and starting HCV treatment with drugs that act directly on the virus under the state drug program for chronic hepatitis C treatment. The patients were hospitalized at the Regional Observational and Infectious Diseases Hospital in Bydgoszcz from 2022 to 2024.

The patients’ medical records were analyzed for cardiovascular risk factors: total cholesterol, LDL (low-density lipoprotein), HDL (high-density lipoprotein), triglycerides, glucose, uric acid, BMI (body mass index), blood pressure, GFR (glomerular filtration rate), CRP (C-reactive protein) and smoking. Also analyzed were the degree of liver fibrosis and steatosis, HCV genotype and the time from the detection of the infection to the inclusion of treatment.

Each patient’s medical history was analyzed for cardiovascular diseases: hypertension, chronic coronary syndrome, myocardial infarction, history of coronary angioplasty, past coronary artery bypass grafting (CABG), stroke or TIA (transient ischemic attack), peripheral vascular disease (carotid atherosclerosis, lower limb atherosclerosis, previous lower limb artery angioplasty and aortic aneurysm), metabolic disease, diabetes and dyslipidemia.

The degree of liver fibrosis and steatosis was assessed by liver elastography performed by fibroscan with the FibroScan Expert 630 and Compact 530 (Echosens, Paris, France) instruments. The assessed parameters were the LSM by VCTE—the liver stiffness measurement by vibration-controlled transient elastography—expressed in kPa and the CAP-controlled attenuation parameter expressed in dB/m. These were respectively converted to the degree of fibrosis and steatosis using Echosens’ validation for HCV infection.

The determination of the HCV RNA and HCV genotype was performed in standardized laboratories by PCR and nucleic acid hybridization methods.

The conducted study had limitations in the comprehensive assessment of the cardiovascular risk in the examined group, as the evaluation of the cardiovascular diseases was based on the medical history collections and the assessment of cardiovascular risk factors through laboratory tests, including blood pressure measurement and BMI. The limitations included the lack of a waist circumference measurement, non-HDL cholesterol levels and apolipoproteins A (apoA) and B (apoB), a carotid artery Doppler ultrasound, coronary artery CT, or coronary angiography to detect the subclinical features of cardiovascular diseases. However, since the patients did not report clinical symptoms, these tests were not performed, and this study was a retrospective review of their medical histories. The medical histories were reviewed for smoking habits, but the use of electronic cigarettes and alcohol consumption were not assessed. Due to the age of the patients, the SCORE—the systematic coronary risk estimation—for assessing cardiovascular death over 10 years and the SCORE2 for assessing the risk of death, myocardial infarction and/or stroke, scales commonly used for this purpose, were not used to assess cardiovascular risk.

### Statistical Methods

The results were presented as the mean ± standard deviation for the quantitative data and as counts expressed in numbers and percentages for the qualitative data. The normal distribution of the data was checked using the Shapiro–Wilk test, while the homogeneity of variance was checked using the Brown–Forsyth test. When the data met the assumptions of parametric analysis for comparing two dependent variables, Student’s *t*-test for the dependent samples and the Wilcoxon paired rank–order test was used when the data did not have a natural distribution and/or a homogeneous variance. Pearson’s chi^2^ test was used to compare abundances. On the other hand, the quantitative data were subjected to a correlation analysis using Pearson’s correlation coefficient for the data with a normal distribution or Spearman’s rank correlation coefficient for the data that did not meet the assumption of normality of distribution. A linear regression and analysis of covariance (ANOVA/ANCOVA) were used in the statistics to account for confounding factors. The level of statistical significance was taken as *p* < 0.05. The statistical analysis was performed using STATISTICA 12.0, StatSoft, Inc. (Tulsa, USA) (2014), www.statsoft.com (StatSoft Poland, Krakow, Poland).

## 3. Results

The study enrolled 217 patients, 96 women and 121 men, representing 42% and 58%, respectively, aged 21 to 45 years (mean age 36 years). Among the subjects, there were 22 patients (10.1%) aged 20–30, 135 (62.2%) aged 30–40 and 60 (27.6%) aged 40–45.

The study group was dominated by genotype 1b (59.45%), genotype 4 (11.52%), genotype 3 (9.68%) and genotype 1a (6.91%), while no genotype was determined (not marked) in 12.44% of the group.

The time from the diagnosis of an HCV infection to the start of treatment in 62.21% of the patients was up to 5 years, between 5 and 10 years in 14.75% and more than 10 years in 18.43%, and in 4.61% of respondents it was not possible to determine the exact time of the detection of the infection.

In the study group, the mean value of liver elasticity was 9.44 kPa (SD = 9.87), corresponding to fibrosis at the F2 level according to the Metavir scale. The level of hepatic steatosis, on the other hand, averaged 222.88 dB/m, corresponding to steatosis at the S0 level according to the Brunt scale.

Based on the analysis of the medical records in the study group, among both men and women, there was no history of burdened cardiovascular diseases: coronary artery disease, myocardial infarction, history of coronary intervention or CABG, stroke or TIA or peripheral artery disease.

Among the subjects, the most common cardiovascular risk factor was cigarette smoking, which was present in 20.7% of the subjects, more often in the age group of 20–30 years and more often in men. Hypertension was present in 12%, in the 20–30 age group in 3 people (13.6%), in the 30–40 age group in 11 people (8.1%) and in the 40–45 age group in 12 people (20%). Hypertension was more common in men.

Diabetes was diagnosed in 5.5% of the patients in the study group, with no cases of diabetes in the 20–30 age range, 3.7% in the 30–40 age range and 11.7% in the 40–45 age range. Diabetes was more common in men. The mean fasting glucose level in the study group was 96.28 mg/dL (SD = 15.69 mg/dL), the mean total cholesterol was 171.09 mg/dL (SD = 36.963 mg/dL), the LDL fraction was 86.32 mg/dL (SD = 26.00 mg/dL), the HDL fraction was 53.24 mg/dL (SD = 16.57 mg/dL) and the TG was 117.49 mg/dL (SD = 51.80 mg/dL).

In the study group, most patients were overweight, with a mean BMI of 26.39 kg/m^2^ (SD = 4.98 kg/m^2^), a minimum of 16.70 and a maximum of 39.91 kg/m^2^.

The mean uric acid value in the study group was 5.14 (SD = 1.52 mg/dL).

The mean concentration of CRP was 1.43 mg/L (SD = 1.81 mg/L). The renal function determined by glomerular filtration rate (GFR) showed an average of 108.46 mL/min/1.73 m^2^ (SD = 23.13 mL/min/1.73 m^2^) (Table 2, Table 3, Table 4 and Table 5).

The study group showed a significant positive relationship between the subjects’ age and glucose. The r-Pearson coefficient r = 0.313, *p* < 0.001 indicates a moderate association in the absolute range |0.3–0.5|, meaning that glucose levels increase with age.

In addition, a significant positive association was found between the subjects’ age and BMI. The r-Pearson coefficient r = 0.188, *p* < 0.006 indicates a weak relationship in the absolute range |0.0–0.3|, meaning that BMI levels increase with age.

There was a significant negative association between the subjects’ age and GFR. The r-Pearson coefficient r = −0.149, *p* < 0.001 indicates a weak association in the absolute range |0.0–0.3|, meaning that GFR levels decrease with age.

In addition, there was a significant positive association between the subjects’ age and liver fibrosis expressed in kPa on the liver elastography by fibroscan. The r-Pearson coefficient r = 0.228, *p* < 0.001 indicates a weak association in the absolute range |0.0–0.3|, indicating that the level of kPA-fibrosis increases with age, and a significant positive association between the age of the subjects and the CAP parameter indicates hepatic steatosis in the fibroscan liver elastography. The r-Pearson coefficient r = 0.211, *p* < 0.025 indicates a weak association in the absolute interval |0.0–0.3|, indicating that CAP increases with age (Table 4).

## 4. Discussion

Recent studies have shown the association of chronic hepatitis C with an increase in the risk of cardiovascular diseases and their complications and a (1.65-fold) increase in the risk of death compared to the uninfected population. The increase in the risk of cardiovascular disease in patients with chronic hepatitis C is 1.75 times greater than in uninfected individuals when an additional risk factor, such as diabetes or hypertension, is present [17,19]. Based on observational studies from Taiwan, HCV has been shown to be an independent factor in stroke and to increase the risk of peripheral arteriosclerosis by 1.43 times. A reduction in the number of acute coronary syndromes and ischemic strokes has been shown in HCV patients treated for cause compared to untreated patients. An American observational study showed that patients with detectable HCV RNA were statistically more likely to have a coronary incident [17,19,25,26]. In the study group of young HCV-infected individuals under 45 years of age, there was no history of cardiovascular disease.

This may most likely be due to the young age of the subjects, which does not increase the risk of cardiovascular disease in the general population, and the low prevalence of other classic modifiable cardiovascular risk factors in the study group.

Patient age (>45 years for men and >55 years for women) is an independent risk factor for hypertension and cardiovascular disease in both the general population and HCV patients; in the study group, the patients were under 45 years old, and the majority of subjects—62.2%—were between 20 and 30 years old. A more advanced age reflects the likelihood of other risk factors. Old age potentially has a longer overlap of single cardiovascular risk factors-more cases of diabetes, hypertension and hyperlipidemia. Also, male gender is a non-modifiable risk factor for cardiovascular disease. In the study group of HCV-infected patients, men accounted for 58% of the subjects; this was unrelated to the increased risk of cardiovascular disease in this group [1,2,8,10].

In the general population, male gender is associated with a 3-fold increased risk of coronary incidents and a 4-fold increased risk of death from coronary causes. This is associated with the presence of other cardiovascular risk factors, such as smoking, elevated levels of total and LDL cholesterol, lower levels of HDL cholesterol and the presence of hypertension. The phenomenon of gender difference is explained by the protective role of estrogens in premenopausal women, estrogens that contribute to the regulation of carbohydrate metabolism, lipid parameters, vascular endothelial function and the homeostatic system. In women with HCV, endocrine disorders are also observed with the progression of liver fibrosis, endocrine disorders, including estrogen production, which are independent of age and may contribute to the occurrence of hypertension in this group of patients. In the group of young subjects analyzed, the women were premenopausal, with little liver fibrosis; the average liver fibrosis was at the F2 level according to Metavir [1,2,3,4].

Based on large Polish population-based surveys (WOBASZ, NATPOL and PolSenior2), it has been shown that hypercholesterolemia may affect 61% of the adult population, hypertension 35%, smoking 26%, diabetes 9% and obesity 22% [1,2,4,6,7,19].

In the study group of HCV-infected young adults, no patient was treated for hyperlipidemia. The mean concentration of total cholesterol in the men was 172.82 mg/dL and of the mean concentration of LDL 88.27 was mg/dL, and in the women the mean concentrations were 168.62 and 83.64 mg/dL, respectively, and they did not meet the criteria for a diagnosis of hypercholesterolemia. In contrast, the average concentration of HDL fraction cholesterol was 51.53 mg/dL in the men and 55.80 mg/dL in the women, which were also normal values. There were no statistically significant differences in the cholesterol levels according to HCV genotype and the duration of the HCV infection. The lower prevalence of lipid disorders in the study group of patients with HCV can be explained by the young age of the patients and the low prevalence of obesity and diabetes among the subjects, as well as the awareness of the application of an appropriate lifestyle, especially dietary recommendations, in patients with liver disease.

Hypertension in the study group occurred in 12% in patients, more often in men, and the frequency increased with age, as in the general population. Although HCV-infected patients have many risk factors that increase the likelihood of hypertension, the study group did not show an increase in its frequency compared to the general population. This may be related to the young age of the subjects, which is associated with a shorter exposure to HCV and thus a shorter time for inflammatory factors to affect the vasculatures, less liver fibrosis and less obesity among the subjects. The development of inflammation appears to be one of the factors contributing to atherosclerosis and vascular damage in HCV. Ceramides composed of long-chain saturated fatty acids contribute to the development of inflammation in HCV patients. The consequences of inflammation are the apoptosis of adipocytes, mobilization of macrophages, formation of inflammatory infiltrate, release of oxygen free radicals, TNF-alpha, FFA and PA I 3, which contribute to the development of hepatic steatosis, insulin resistance, obesity and consequently the development of atherosclerosis and hypertension. TNF-alpha deregulates post-receptor pathways for insulin, blocks the insulin receptor connection to IRS-1, inhibits phosphatidyl-inositol-3 kinase and deregulates glycogen, fat and protein synthesis. IL-6 reduces insulin receptor autophosphorylation and IRS-2 phosphorylation. Oxidative stress also appears to contribute to metabolic dysfunction, resulting in the development of atherosclerosis and increased blood pressure in HCV patients. It can result in insulin resistance due to the activation of the protein kinase c-JUN N-terminal kinase, which causes IRS degradation [17,19,23,27,28,29,30].

Also, a higher risk of developing hypertension is observed with the severity of liver fibrosis; in the study group of patients, liver fibrosis was insignificant and rated at an average of F2 according to Metavir [17,19,25]. The effect of HCV on the activity of cytokines produced by adipose tissue and the associated weight gain and development of obesity may be responsible for the increased incidence of hypertension in HCV patients.

HCV patients have reduced levels of adiponectin and leptin, and increased levels of resistin, ghrelin and vizfatin, contributing to inflammation, insulin resistance, lipid disorders and atherosclerosis and consequently to the development of hypertension and obesity [8,21,22].

In the general population of Poland, obesity occurs in 22% of people, while in the population of HCV patients, obesity occurs in 17–38% of patients; this is related to the abnormalities of the adipocytokines produced by the adipose tissue mentioned above [1,6,31,32,33,34].

In the study group, the average body mass index BMI was 26.39 kg/m^2^; in the women, it was 24.61, and in the men, it was 27.77 kg/m^2^. The BMI increased with age. However, there were no statistically significant differences related to HCV genotype or length of infection in the young adults.

Type 2 diabetes occurs in 9% of Polish adults [1,6,35,36]. Epidemiological data show an association between HCV infection and the development of carbohydrate disorders, including abnormal fasting glucose, glucose intolerance or diabetes. It is estimated that the prevalence of carbohydrate disorders in patients with chronic hepatitis C is four to ten times higher than in the population of healthy people and occurs in 14–30% of patients. Hence, HCV infection is now considered a risk factor for the development of diabetes, and diabetes is considered an extrahepatic manifestation of HCV infection. Based on long-term observations, it has been shown that risk factors for the development of diabetes in patients with HCV are older age, HCV genotype 3, markedly severe fibrosis or cirrhosis, a positive family history of diabetes and kidney or liver transplantation [8,20,22,37,38,39,40]. The increased risk of developing diabetes in HCV infections is associated with insulin resistance and a chronic inflammatory response due to an increased synthesis of pro-inflammatory cytokines, mainly tumor necrosis factor (TNF) alpha and interleukin-6 (IL-6) [27,41,42]. As a result of insulin resistance, there is an increase in the concentration of insulin, which is not used efficiently by tissues, and secondary hyperinsulinemia, followed by an increase in serum glucose and the development of carbohydrate disorders. Insulin resistance also enhances adipose tissue lipolysis and the availability of free fatty acids, consequently leading to hepatic steatosis. The phenomenon of insulin resistance affects all HCV genotypes; however, it is most pronounced in genotype 3 and coexists with a lower HOMA index value [20,38,40].

The development of insulin resistance has also been linked to the direct effects of the virus on the pathway insulin signaling. In HCV genotype 3, viral proteins can directly affect intrahepatic insulin signaling by downregulating the expression of the peroxisome proliferator-activated receptor alpha (PPAR alpha). It controls the gene expression of mitochondrial carnitine palmitoyltransferase-1 (CPT-1), which reduces mitochondrial beta-oxidation responsible for fatty acid catabolism and acetyl CoA oxidase (AOX). One mechanism of insulin resistance in patients with chronic liver disease is acquired growth hormone (GH-growth hormone) resistance, which is caused by an increase in pro-inflammatory cytokines, mainly TNF alpha. Acquired GH resistance consequently causes a decrease in insulin-like growth factor-1 (IGF-1) and a compensatory increase in GH, exacerbating insulin resistance secondarily [42,43,44,45]. Insulin resistance through the increased influx of free fatty acids (FFA) into the liver, hypertriglyceridemia and hyperinsulinemia exacerbates hepatic steatosis and fibrosis. Recent studies have shown that a decreased insulin receptor substrate-1 (IRS-1 insulin receptor substrate-1) and kinase B (PKB/Akt) phosphorylation plays an important role in the pathomechanism of insulinoproneness in HCV patients. In addition, HCV affects the insulin signaling pathway through SOCS-3 (suppressor of cytokines signaling proteins) regulatory proteins, which are directly stimulated by HCV core proteins and cause the degradation of IRS-1 AND IRS-2, inhibiting relay in the insulin signaling pathway. Chronic inflammation occurring in HCV infections is also associated with the increased expression of SOCS-1 and SOCS-3 in the liver, which is associated with a decreased response to antiviral treatment. SOCS-3 also stimulates the expression of sterol regulatory element response sequence binding protein 1 c (SREBP-1c), associated with fatty acid metabolism in the liver, causing steatosis. In HCV genotype 3, core proteins enhance the degradation of IRS-1 by the aforementioned reduction in peroxisome proliferator-activated receptor gamma- (PPAR-gamma) synthesis and stimulation of SOCS-7 regulatory protein synthesis. Insulin resistance is also associated with increased levels of pro-inflammatory cytokines, TNF alpha and IL-6 [46]. The presence of insulin resistance in metabolic steatosis is correlated with its severity and appears to be an unfavorable determinant of fibrosis progression. An increased risk of developing diabetes is also associated with increased leptin secretion, resistin and decreased adiponectin secretion by adipose tissue [22,25,39].

The mean glucose level in the study group of the young adults infected with HCV was 96.28 mg/dL, and glucose levels were shown to increase with age. In contrast, in the study group of the young HCV patients, the prevalence of diabetes was 5.5% and increased with age. There were no statistically significant differences according to HCV genotype as well as length of infection.

Another cardiovascular disease risk factor analyzed was cigarette smoking. In Poland, 26% of the adult population smokes cigarettes [1,6,47]. In the study group, 20.7% of the subjects smoked, more often men. The lower number of cigarette smokers in the study group may be due to awareness of the harmful effects of smoking on the body in HCV patients and the conscious cessation of smoking, as well as the decreasing prevalence of cigarette smoking in society, especially among young people, observed in the last several years [47,48,49].

Uric acid levels were also analyzed, which averaged 5.14 mg/dL and did not depend on HCV genotype or the duration of infection. It was also within the sex-specific normal range [1,50,51,52].

In the study group of HCV-infected patients under the age of 45, there was also no increased number of patients with chronic kidney disease as a cardiovascular risk factor, and the average glomerular filtration rate (GFR) was 108, 46 mL/min. This may be due to the young age of the patients, as well as the small number of patients in the study group with diabetes or hypertension. Renal extrahepatic manifestations of HCV infection such as glomerulonephritis were also not observed in the study group [1,3,24].

Studies in recent years have highlighted the role of inflammation, particularly low-grade chronic inflammation, in the pathogenesis of coronary artery disease. C-reactive protein is an inflammatory molecule that has demonstrated value as a predictive marker in cardiovascular risk assessments, both independently and in combination with other parameters. CRP, which is mainly synthesized in the liver under the influence of pro-inflammatory cytokines such as IL-6, IL-1β and TNF, plays a key role in the progression of atherosclerotic cardiovascular disease.

Most studies have shown no or a statistically insignificant impact of HCV infection on CRP levels [53]. Based on available studies determining the relationship between HCV infection and CRP levels, higher CRP values are observed in individuals with detectable HCV- RNA viremia compared to those with a negative HCV- RNA status, and higher levels are observed in individuals infected with HCV genotype 2 compared to genotypes 1 and 3, although this was not statistically significant [54]. However, other studies have observed that IL-6 levels, which play an important role in regulating the inflammatory response and stimulate CRP production, decrease in HCV infections, explaining this through the mechanism of so-called immune tolerance due to the continuous replication of the hepatitis C virus [55,56].

In the study group of HCV-infected young adults, the mean C-reactive protein value was within normal limits, averaging 1.43 mg/L, but in terms of cardiovascular risk, values > 1 suggest a moderate cardiovascular risk. Based on available studies, CRP values below 1 correspond to a low cardiovascular risk, while values above 3 correspond to a high risk [57,58,59,60].

Based on the analysis, there was no increased risk of cardiovascular disease in the population of young adults under 45 years of age with chronic hepatitis C, and the prevalence of individual risk factors was comparable or lower than in the general population. To date, HCV infection has been considered a new, non-classical risk factor for cardiovascular disease, and cardiovascular disease an extrahepatic manifestation of HCV, but most of the studies supporting this thesis have been in the population of adults aged 45 and older [17,19,22,26]. The different results obtained in this study, compared to other researchers, related to the lack of an increase in cardiovascular morbidity among young HCV patients, may indicate that the most important risk factor for cardiovascular disease in HCV-infected patients is the patient’s age and the long duration of an HCV infection, which is associated with the likelihood of other cardiovascular factors with age, including diabetes, hypertension, obesity and more severe liver fibrosis. In addition, in recent years, and especially among young people and young adults, there has been a noticeable shift to more health-promoting lifestyles, paying attention to healthy diets and regular physical activity, and a turn away from smoking, compared to the older generation.

These obtained results require further research and can be considered as a pilot study.

These results should encourage the testing of young people for HCV infection, which will contribute to rapid detection and treatment and may help reduce the incidence of cardiovascular disease in this population [25,26,61,62].

## 5. Conclusions

1. HCV does not increase cardiovascular risk in young adults up to the age 45.
2. The prevalence of cardiovascular risk factors in young adults infected with HCV is not increasing.
3. HCV genotype has no effect on the prevalence of cardiovascular factors in young adults.
4. HCV infection may cause an increase in CRP levels, which is associated with an increased cardiovascular risk, but this requires further research.
5. Age, the duration of HCV infection and the severity of liver fibrosis appear to be the most significant risk factors for cardiovascular disease in HCV-infected patients.
6. The early detection of HCV infection and its early treatment can provide the prevention of cardiovascular disease.

## Figures and Tables

**Table 1:** Classic risk factors for cardiovascular disease.

Modifiable Factors	Non-Modifiable Factors
Improper diet; Smoking; Reduced physical activity; Hypertension; Increased serum levels of total cholesterol, LDL fraction and triglycerides; Reduced serum levels of HDL fraction cholesterol; Diabetes and prediabetic condition Obesity; Metabolic syndrome; Chronic kidney disease; Increased serum levels of C-reactive protein, fibrinogen and homocysteine.	Age ≥ 45 in men and ≥55 in women; Male gender; A loaded family history in men < 55 years of age and in women < 65 years of age; History of cardiovascular disease; Genetic factors.

**Table 2:** Total results. (1) Total results by gender. (2) Results by age group. (3) Results by age group and gender.

					Average	Median	Standard Deviation	Minimum	Maximum
**Total cholesterol mg/dL**					171.09	174.00	36.963	85.00	265.00
LDL mg/dL					86.31	85.50	26.01	48.00	133.00
HDL mg/dL					53.24	53.00	16.57	26.00	103.00
TG mg/dL					117.49	110.00	51.80	47.00	260.00
CRP mg/L					1.43	0.70	1.81	0.00	16.20
Uric acid mg/dL					5.14	4.80	1.52	2.00	10.90
GFR mL/min/1.73 m^2^					108.46	106.00	23.13	24.00	232.00
LSM by VCTE (kPa)					9.44	6.10	9.87	2.80	74.60
CAP (dB/m)					222.88	217.00	49.03	105.00	400.00
BMI kg/m^2^					26.39	25.70	4.98	16.70	39.91
Glucose mg/dL					96.28	95.00	15.69	69.00	199.00
**(1)**									
**Gender M/W**					**Total Cholesterol mg/dL**	**LDL mg/dL**	**HDL mg/dL**	**TG mg/dL**	**CRP mg/L**
Women	N	96							
	Average				168.66	83.64	55.80	108.94	0.96
	Median				167.00	77.00	54.50	100.50	0.59
	Standard deviation				39.68	27.44	17.643	42.19	0.82
	Minimum				106.00	51.00	32.00	47.00	0.00
	Maximum				265.00	125.00	103.00	260.00	5.80
Men	N	121							
	Average				172.82	88.27	51.53	124.17	1.75
	Median				176.00	91.00	47.00	111.00	0.90
	Standard deviation				35.26	25.70	15.78	57.84	2.20
	Minimum				85.00	48.00	26.00	50.00	0.60
	Maximum				260.00	133.00	89.00	256.00	16.20
Statistics									
**Gender M/W**			**Uric Acid mg/dL**		**GFR mL/min/1.73 m^2^**	**LSM by VCTE** **kPa**	**CAP** **dB/m**	**BMI kg/m^2^**	**Glucose mg/dL**
Women	Average		4.25		106.09	7.190	214.61	24.60	93.70
	Median		4.40		103.00	5.75	203.00	23.10	94.00
	Standard deviation		0.94		21.14	4.29	51.88	5.02	10.40
	Minimum		2.00		24	3.00	105.00	16.70	70.00
	Maximum		7.10		169	23.50	400.00	38.28	126.00
Men	Average		5.87		110.36	11.22	229.69	27.77	98.32
	Median		6.00		106.00	6.50	228.50	27.40	95.00
	Standard deviation		1.53		24.528	12.39	45.87	4.509	18.65
	Minimum		2.00		64.00	2.80	144.00	18.50	69.00
	Maximum		10.90		232.00	74.60	333.00	39.91	199.00
**(2)**									
**Age in Ranges**			**Uric Acid** **mg/dL**		**GFR** **mL/min/1.73 m^2^**	**LSM by VCTE** **(kPa)**	**CAP (dB/m)**	**BMI** **kg/m^2^**	**Glucose** **mg/dL**
Age 20 to 30	N	22							
	Average		5.85		113.76	7.06	216.60	25.49	88.05
	Median		5.00		114.00	5.55	208.00	25.80	87.00
	Standard deviation		2.28		17.12	4.75	53.63	4.55	8.72
	Minimum		3.90		85.00	3.80	105.00	18.70	70.00
	Maximum		10.90		149.00	22.70	301.00	35.80	105.00
Age 30 to 40	N	135	72.00		135.00	135.00	70.00	131.00	135.00
	Average		5.06		108.98	8.51	217.96	26.08	94.35
	Median		4.75		106.00	5.80	215.00	25.50	93.00
	Standard deviation		1.44		21.88	8.97	44.17	4.88	13.80
	Minimum		2.00		69.00	3.50	137.00	16.70	69.00
	Maximum		9.10		232	74.60	294.00	38.80	195.00
Age 40 to 45	N	60							
	Average		5.13		105.45	12.40	238.57	27.38	103.63
	Median		5.45		103.00	7.07	232.00	25.90	98.50
	Standard deviation		1.47		27.26	12.36	56.17	5.26	18.78
	Minimum		2.20		24.00	2.80	144.00	18.87	85.00
	Maximum		7.70		207.00	72.60	400.00	39.91	199.00
**Age in Ranges**					**Total Cholesterol** **mg/dL**	**LDL** **mg/dL**	**HDL** **mg/dL**	**TG** **mg/dL**	**CRP** **mg/L**
Age 20 to 30	Average				149.56	70.33	48.22	90.78	3.159
	Median				146.00	67.00	46.00	78.00	1.400
	Standard deviation				22.28	21.20	15.71	28.25	4.78
	Minimum				121.00	51.00	26.00	54.00	0.60
	Maximum				178.00	93.00	69.00	123.00	16.20
Age 30 to 40	Average				177.89	91.14	55.95	121.41	1.257
	Median				178.00	95.50	55.00	106.00	0.590
	Standard deviation				40.47	28.85	16.93	53.77	1.191
	Minimum				85.00	48.00	27.00	47.00	0.60
	Maximum				265.00	133.00	103.00	260.00	5.80
Age 40 to 45	Average				166.71	84.11	50.13	120.96	1.34
	Median				170.50	80.00	45.00	114.00	0.800
	Standard deviation				31.46	22.52	15.86	53.85	1.35
	Minimum				106.00	57.00	32.00	56.00	0.00
	Maximum				230.00	118.00	78.00	256.00	6.60
**(3)**									
**Age in Ranges**	**Gender M/F**				**Total Cholesterol**	**LDL**	**HDL**	**TG**	**CRP**
Age 20 to 30	Women		N	10					
			Average		151.00	67.00	50.33	89.67	1.04
			Median		146.00	67.00	54.00	78.00	1.20
			Standard deviation		22.91		15.82	29.30	0.4152
			Minimum		131	67	33	68	0.60
			Maximum		176	67	64	123	1.40
	Men		N	12					
			Average		148.83	72.00	47.17	91.33	5.282
			Median		148.50	72.00	45.00	91.50	2.600
			Standard deviation		24.13	29.70	17.06	30.54	6.32
			Minimum		121.00	51.00	26.00	54.00	0.60
			Maximum		178.00	93.00	69.00	123.00	16.20
Age 30 to 40	Women		N	65					
			Average		175.24	86.00	58.47	110.05	0.92
			Median		174.00	91.00	55.00	95.00	0.59
			Standard deviation		42.45	34.18	19.23	46.77	0.923
			Minimum		120.00	51.00	34.00	47.00	0.60
			Maximum		265.00	125.00	103.00	260.00	5.80
	Men		N	70					
			Average		180.30	94.00	53.96	133.35	1.52
			Median		178.00	100.00	55.50	128.00	0.750
			Standard deviation		39.38	27.25	14.99	59.09	1.32
			Minimum		85.00	48.00	27.00	50.00	0.60
			Maximum		260.0	133.00	89.00	256.00	4.70
Age 40 to 45	Women		N	21					
			Average		158.00	84.60	51.50	113.25	1.01
			Median		158.50	77.00	54.50	114.50	0.59
			Standard deviation		35.81	25.24	14.61	34.718	0.64
			Minimum		106.00	57.00	32.00	61.00	0.00
			Maximum		210.00	118.00	74.00	156.00	2.2
	Men		N	37					
			Average		171.06	83.50	49.40	125.07	1.49
			Median		174.00	80.00	43.00	111.00	0.85
			Standard deviation		29.29	22.41	16.94	62.46	1.56
			Minimum		116.00	60.00	33.00	56.00	0.60
			Maximum		230.00	114.00	78.00	256.00	6.60

BMI, body mass index; CAP, controlled attenuation parameter; CRP, C-reactive protein; GFR, glomerular filtration rate; HDL, high-density lipoprotein; LDL, low-density lipoprotein; LSM by VCTE, liver stiffness measurement by vibration-controlled transient elastography; TG, triglycerides.

**Table 3:** Results by sex, time of infection and HCV genotype.

Gender M/F	HCV Genotype	Time of Infection Years				Total Cholesterol mg/dL		LDL mg/dL		HDL mg/dL		TG mg/dL		CRP mg/L
**Women**	No data available	Up to 5 years	N 12											
			Average			199.25				64.67		125.75		0.59
			Median			199.00				65.00		128.50		0.59
			Standard deviation			25.73				0.58		27.50		0.00
			Minimum			176.00				64.00		90.00		0.60
			Maximum			223.00				65.00		156		0.60
		Between 5 and 10 years	N 0			0		0		0		0		0
		Over 10 years	N 0			0		0		0		0		0
	1a	Up to 5 years	N 4											
			Average			148.00				56.00		156.00		0.99
			Median			148.00				56.00		156.00		0.99
			Standard deviation											0.57
			Minimum			148.00				56.00		156.00		0.60
			Maximum			148.00				56.00		156.00		1.40
		Between 5 and 10 years	N 2											
			Average			131.00		67.00		54.00		68.00		
			Median			131.00		67.00		54.00		68.00		
			Minimum			131.00		67.00		54.00		68.00		
			Maximum			131.00		67.00		54.00		68.00		
		Over 10 years	N 4											
			Average			215.00				44.00		121.50		0.59
			Median			215.00				44.00		121.50		0.59
			Standard deviation			70.71				0.00		48.79		
			Minimum			165.00				44.00		87.00		0.60
			Maximum			265.00				44.00		156.00		0.60
	1b	Up to 5 years	N 32			9								
			Average			165.33		108.00		57.67		127.89		0.86
			Median			156.00		115.00		46.00		115.00		0.59
			Standard deviation			32.14		21.34		20.92		56.95		0.43
			Minimum			128.00		77.00		32.00		62.00		0.60
			Maximum			227.00		125.00		88.00		260.00		2.00
		Between 5 and 10 years	N 8											
			Average			158.50		103.00		58.50		96.50		0.47
			Median			158.50		103.00		58.50		96.50		0.59
			Standard deviation			45.96				21.92		19.09		0.32
			Minimum			126.00		103.00		43.00		83.00		0.00
			Maximum			191.00		103.00		74.00		110.00		0.70
		Over 10 years	N 9											
			Average			210.00				52.50		104.50		1.24
			Median			210.00				52.50		104.50		1.40
			Standard deviation			0.00				4.95		54.45		0.674
			Minimum			210.00				49.00		66.00		0.60
			Maximum			210.00				56.00		143.00		2.20
	3	Up to 5 years	N 6											
			Average			137.00		72.00		63.67		80.33		0.84
			Median			130.00		68.00		53.00		85.00		0.84
			Standard deviation			35.03		17.35		35.23		17.47		0.36
			Minimum			106.00		57.00		35.00		61.00		0.60
			Maximum			175.00		91.00		103.00		95.00		1.10
		Between 5 and 10 years	N 0			0		0		0		0		1
		Over 10 years	N 2											
			Average			126.00				38.00		74.00		1.40
			Median			126.00				38.00		74.00		1.40
			Minimum			126.00				38.00		74.00		1.40
			Maximum			126.00				38.00		74.00		1.40
	4	Up to 5 years	N 7											
			Average			146.33		51.00		66.00		94.00		0.59
			Median			145.00		51.00		66.00		90.00		0.59
			Standard deviation			27.02				15.56		49.12		0.00
			Minimum			120.00		51.00		55.00		47.00		0.60
			Maximum			174.00		51.00		77.00		145.00		0.60
		Over 10 years	N 5											
			Average			166.67		51.00		40.67		81.33		2.09
			Median			146.00		51.00		34.00		78.00		0.99
			Standard deviation			59.74				12.42		7.57		2.50
			Minimum			120.00		51.00		33.00		76.00		0.60
			Maximum			234.00		51.00		55.00		90.00		5.8
	indefinite	Between 5 and 10 years	N 0											
Men		Up to 5 years	N 3											
			Average			121.00				33.00		122.00		3.20
			Median			121.00				33.00		122.00		2.10
			Standard deviation											2.08
			Minimum			121.00				33.00		122.00		1.90
			Maximum			121.00				33.00		122.00		5.60
		Over 10 years	N 2											
			Average			140.00		74.00		54.00		77.00		1.64
			Median			140.00		74.00		54.00		77.00		1.64
			Standard deviation			33.94		36.77		9.90		38.18		1.49
			Minimum			116.00		48.00		47.00		50.00		0.60
			Maximum			164.00		100.00		61.00		104.00		2.70
	1	Up to 5 years	N 0											
	1a	Up to 5 years	N 1											
			Average			124.00		51.00		65.00		54.00		0.59
			Median			124.00		51.00		65.00		54.00		0.59
			Minimum			124.00		51.00		65.00		54.00		0.60
			Maximum			124.00		51.00		65.00		54.00		0.60
		Between 5 and 10 years	N 4											
			Average			161.50		114.00		39.00		115.50		3.44
			Median			161.50		114.00		39.00		115.50		4.10
			Standard deviation			9.19				7.07		7.78		2.09
			Minimum			155.00		114.00		34.00		110.00		1.10
			Maximum			168.00		114.00		44.00		121.00		5.10
		Over 10 years	N 1											
			Average			192.00				33.00		234.00		1.20
			Median			192.00				33.00		234.00		1.20
			Minimum			192.00				33.00		234.00		1.20
			Maximum			192.00				33.00		234.00		1.20
	1b	Up to 5 years	N 51											
			Average			180.75		103.00		52.75		101.46		1.46
			Median			178.00		112.00		56.00		79.00		0.60
			Standard deviation			36.24		35.37		14.09		55.67		1.49
			Minimum			116.00		64.00		27.00		55.00		0.60
			Maximum			260.00		133.00		75.00		256.00		6.60
		Between 5 and 10 years	N 11											
			Average			185.00		80.00		56.00		175.00		0.95
			Median			187.00		80.00		55.50		171.50		0.70
			Standard deviation			4.69				9.49		56.69		0.55
			Minimum			178.00		80.00		45.00		111.00		0.60
			Maximum			188.00		80.00		68.00		246.00		2.00
		Over 10 years	N 17											
			Average			168.00		90.50		49.71		150.67		1.587
			Median			168.00		90.50		40.00		143.00		0.950
			Standard deviation			48.05		21.92		19.56		59.69		1.33
			Minimum			85.00		75.00		33.00		94.00		0.60
			Maximum			230.00		106.00		78.00		256.00		4.30
	3	Up to 5 years	N 11											
			Average			169.50		75.50		48.00		126.75		1.70
			Median			172.50		75.50		43.50		126.50		0.80
			Standard deviation			41.62		21.92		12.94		52.18		1.52
			Minimum			116.00		60.00		38.00		65.00		0.60
			Maximum			217.00		91.00		67.00		189.00		4.70
		Between 5 and 10 years	N 2											
			Average			162.00		80.00		52.00		190.00		1.19
			Median			162.00		80.00		52.00		190.00		1.19
			Standard deviation											0.86
			Minimum			162.00		80.00		52.00		190.00		0.60
			Maximum			162.00		80.00		52.00		190.00		1.80
	4	Up to 5 years	N 8											
			Average			184.50		117.00		59.75		131.25		4.39
			Median			181.00		117.00		62.00		139.50		.59
			Standard deviation			37.85				28.96		49.38		6.76
			Minimum			142.00		117.00		26.00		66.00		0.60
			Maximum			234.00		117.00		89.00		180.00		16.20
		Between 5 and 10 years	N 5											
			Average			173.00		93.00		69.00		73.00		0.80
			Median			173.00		93.00		69.00		73.00		0.80
			Standard deviation											0.14
			Minimum			173.00		93.00		69.00		73.00		0.70
			Maximum			173.00		93.00		69.00		73.00		0.90
		Over 10 years	N 0											
	-	Up to 5 years	N 1											
			Average			178.00				34.00		78.00		1.30
			Median			178.00				34.00		78.00		1.30
			Minimum			178.00				34.00		78.00		1.30
			Maximum			178.00				34.00		78.00		1.30
**Gender M/W**	**HCV Genotype**	**Time of Infection**			**Uric Acid**	**GFR**		**Fibroscan or SWE (kPa)**		**Fibroscan (CAP)**		**BMI**		**Glucose**
**Women**		Up to 5 years	N 11											
			Average		4.20	99.00		6.62		233.78		25.21		90.42
			Median		4.40	99.00		5.45		201.00		22.20		89.00
			Standard deviation		0.71	11.64		4.36		85.91		6.82		13.65
			Minimum		3.00	78.00		4.10		105.00		16.70		70.00
			Maximum		4.80	121.00		20.00		400.00		37.24		126.00
		Between 5 and 10 years	N 1											
			Average		3.90	115.00		5.30		194.00		18.70		90.00
			Median		3.90	115.00		5.30		194.00		18.70		90.00
			Minimum		3.90	115.00		5.30		194.0		18.70		90.00
			Maximum		3.90	115.00		5.30		194.0		18.70		90.00
		Over 10 years	N 1											
			Average			80.00		6.20		149.00		20.03		88.00
			Median			80.00		6.20		149.00		20.03		88.00
			Minimum			80.00		6.20		149.00		20.03		88.00
			Maximum			80.00		6.20		149.00		20.03		88.00
	1	Over 10 years	N 1											
			Average		5.20	101.00		5.30		203.00		23.20		84.00
			Median		5.20	101.00		5.30		203.00		23.20		84.00
			Minimum		5.20	101.00		5.30		203.0		23.20		84.00
			Maximum		5.20	101.00		5.30		203.0		23.20		84.00
	1a	Up to 5 years	N 4											
			Average		4.33	108.75		5.87		221.00		21.27		95.50
			Median		4.60	115.50		5.60		221.00		19.23		96.00
			Standard deviation		0.64	21.41		1.50		72.12		4.23		8.54
			Minimum		3.60	78.00		4.60		170.0		19.00		85.00
			Maximum		4.80	126.00		7.70		272.00		27.60		105.00
		Between 5 and 10 years	N 2											
			Average			132.50		14.10		227.00		23.35		89.50
			Median			132.50		14.10		227.00		23.35		89.50
			Standard deviation			17.68		12.162				3.46		3.54
			Minimum			120.00		5.50		227.0		20.90		87.00
			Maximum			145.00		22.70		227.0		25.80		92.00
		Over 10 years	N 4											
			Average		3.800	112.00		6.2750		238.000		23.9975		100.25
			Median		3.80	109.00		6.25		238.00		24.79		103.00
			Standard deviation			18.46		1.13		5.66		2.90		17.21
			Minimum		3.80	93.00		5.30		234.0		19.83		78.00
			Maximum		3.80	137.00		7.30		242.0		26.57		117.00
	1b	Up to 5 years	N 32											
			Average		4.46	104.72		6.70		195.47		24.20		94.22
			Median		4.40	100.50		5.60		198.00		23.15		95.00
			Standard deviation		1.156	26.92		3.48		37.89		3.63		12.22
			Minimum		2.00	24.00		3.50		137.0		17.30		72.00
			Maximum		7.10	169.00		18.10		264.00		31.20		126.00
		Between 5 and 10 years	N 8											
			Average		3.50	106.25		6.62		185.00		25.49		92.50
			Median		3.45	101.50		5.70		185.00		22.83		88.50
			Standard deviation		0.71	20.19		2.60		31.11		6.71		9.05
			Minimum		2.70	83.00		3.80		163.00		18.87		84
			Maximum		4.40	141.00		11.80		207.0		38.28		111.00
		Over 10 years	N 9											
			Average		3.90	107.56		6.80		239.50		28.47		96.78
			Median		4.40	108.00		6.10		259.00		30.24		96.00
			Standard deviation		1.51	12.65		1.60		39.36		5.78		5.89
			Minimum		2.20	90.00		5.30		180.00		20.20		87.00
			Maximum		5.10	134.00		9.60		272.00		35.20		105.00
	3	Up to 5 years	N 6											
			Average		4.65	109.17		11.44		249.50		25.43		98.50
			Median		4.65	109.50		6.67		249.50		24.40		98.00
			Standard deviation		0.93	33.87		9.25		54.45		4.95		3.89
			Minimum		3.60	71.00		3.00		211.00		19.50		94.00
			Maximum		5.70	162.00		23.50		288.0		33.85		103.00
		Between 5 and 10 years	N 1											
			Average			90.00		12.40				38.00		101.00
			Median			90.00		12.40				38.00		101.00
			Minimum			90.00		12.40				38.00		101.00
			Maximum			90.00		12.40				38.00		101.00
		Over 10 years	N 2											
			Average		4.60	112.50		4.65				25.22		93.50
			Median		4.60	112.50		4.65				25.22		93.50
			Standard deviation			0.71		0.78				6.40		7.78
			Minimum		4.60	112.00		4.10				20.70		88.00
			Maximum		4.60	113.00		5.20				29.75		99.00
	4	Up to 5 years	N 7											
			Average		4.27	107.00		9.33		214.75		23.34		91.00
			Median		4.50	109.00		6.40		204.50		20.75		89.00
			Standard deviation		0.49	15.25		5.42		37.43		5.36		8.08
			Minimum		3.7	84.00		5.20		184.00		18.43		83.00
			Maximum		4.6	128.00		18.40		266.00		31.24		107.00
		Over 10 years	N 5											
			Average		3.47	109.00		5.22		228.50		22.29		91.60
			Median		3.50	103.00		4.60		228.50		20.70		90.00
			Standard deviation		0.25	14.78		0.95		85.56		3.64		6.58
			Minimum		3.20	92.00		4.40		168.00		18.07		83.00
			Maximum		3.70	126.00		6.40		289.00		26.70		101.00
	Indefinite	Between 5 and 10 years	N 1											
			Average		5.30	120.00		6.90		205.00		22.85		86.00
			Median		5.30	120.00		6.90		205.00		22.85		86.00
			Minimum		5.30	120.00		6.90		205.00		22.85		86.00
			Maximum		5.30	120.00		6.90		205.00		22.85		86.00
Men		Up to 5 years	N 3											
			Average		7.70	111.33		21.57		266.00		29.30		100.33
			Median		6.10	108.00		20.20		266.00		27.40		96.00
			Standard deviation		2.77	26.16		2.542		45.25		5.79		9.29
			Minimum		6.10	87.00		20.00		234.00		24.70		94.00
			Maximum		10.90	139.00		24.50		298.00		35.80		111.00
		Over 10 years	N 2											
			Average		7.50	105.50		8.37		253.50		24.02		96.50
			Median		7.50	105.50		8.37		253.50		24.02		96.50
			Standard deviation			44.55		3.44		45.96		4.42		16.26
			Minimum		7.50	74.00		5.94		221.0		20.90		85.00
			Maximum		7.50	137.00		10.80		286.0		27.15		108.00
	1	Up to 5 years	N 1											1=
			Average			121.00		7.80						78.00
			Median			121.00		7.80						78.00
			Minimum			121.00		7.80						78.00
			Maximum			121.00		7.80						78.00
	1a	Up to 5 years	N 1											
			Average			114.00		7.00		208.00		23.40		101.00
			Median			114.00		7.70		208.00		23.40		101.00
			Minimum			114.00		7.70		208.00		23.40		101.00
			Maximum			114.00		7.70		208.00		23.40		101.00
		Between 5 and 10 years	N 4											
			Average		6.70	111.50		5.62		229.00		28.46		91.00
			Median		6.70	109.00		5.45		229.00		28.15		93.50
			Standard deviation			15.97		0.64				2.81		7.62
			Minimum		6.70	95.00		5.10		229.00		25.40		80.00
			Maximum		6.70	133.00		6.50		229.00		32.14		97.00
		Over 10 years	N 1											
			Average		6.10	157.00		5.60				31.49		86.00
			Median		6.10	157.00		5.60				31.49		86.00
			Minimum		6.10	157.00		5.60				31.49		86.00
			Maximum		6.10	157.00		5.60				31.49		86.00
	1b	Up to 5 years	N 51											
			Average		5.51	110.63		11.19		221.07		26.90		99.37
			Median		5.50	106.00		6.40		212.00		25.73		97.00
			Standard deviation		1.34	29.12		12.58		48.15		4.17		18.42
			Minimum		3.00	64.00		3.80		144.0		18.50		76.00
			Maximum		9.10	232.00		74.60		333.0		38.80		195.00
		Between 5 and 10 years	N 11											
			Average		6.85	105.82		8.95		244.75		29.85		111.27
			Median		7.20	101.00		5.90		256.00		30.00		97.00
			Standard deviation		1.58	21.53		7.24		46.03		5.41		36.17
			Minimum		4.20	82.00		3.80		181.00		22.89		69.00
			Maximum		8.80	147.00		26.10		286.00		39.91		199.00
		Over 10 years	N 17											
			Average		5.64	108.06		12.08		246.00		28.53		96.41
			Median		5.95	107.00		6.60		250.00		28.69		91.00
			Standard deviation		1.29	17.26		11.04		46.80		5.190		14.74
			Minimum		3.80	76.00		2.80		156.00		20.00		85.00
			Maximum		7.70	145.00		34.60		304.00		37.04		146.00
	3	Up to 5 years	N 11											
			Average		6.40	104.55		17.05		223.12		29.06		94.73
			Median		6.40	107.00		6.40		234.50		28.50		91.00
			Standard deviation		0.14	19.53		22.54		42.51		4.56		13.42
			Minimum		6.30	80.00		5.10		155.00		21.36		80.00
			Maximum		6.50	148.00		72.60		265.00		36.80		124.00
		Between 5 and 10 years	N 2											
			Average		4.90	102.50		9.850				32.00		95.50
			Median		4.90	102.50		9.85				32.00		95.50
			Standard deviation			17.68		2.90				8.48		0.71
			Minimum		4.90	90.00		7.80				26.00		95.00
			Maximum		4.90	115.00		11.90				38.00		96.00
	4	Up to 5 years	N 8											
			Average		4.15	103.00		5.59		223.00		25.37		91.88
			Median		4.15	103.00		5.10		208.00		25.58		87.00
			Standard deviation		3.04	10.02		1.33		52.14		2.96		15.60
			Minimum		2.00	85.00		4.80		180.00		21.79		74.00
			Maximum		6.30	118.00		8.80		281.00		31.00		126.00
		Between 5 and 10 years	N 5											
			Average		5.97	129.60		14.72		227.33		26.33		94.40
			Median		6.15	124.00		6.50		213.00		25.80		94.00
			Standard deviation		1.09	30.81		19.65		67.65		3.61		3.65
			Minimum		4.50	99.00		4.10		168.00		22.60		90.00
			Maximum		7.10	172.00		49.70		301.00		30.22		99.00
		Over 10 years	N 3											
			Average		5.30	124.00		6.93		283.00		31.41		103.33
			Median		5.30	132.00		7.40		283.00		31.00		103.00
			Standard deviation			23.06		2.93				1.73		17.50
			Minimum		5.30	98.00		3.80		283.0		29.92		86
			Maximum		5.30	142.00		9.60		283.00		33.30		121.00
	-	Up to 5 years	N 1											
			Average			121.00		12.10		234.00		30.22		93.00
			Median			121.00		12.10		234.00		30.22		93.00
			Minimum			121.00		12.10		234.00		30.22		93.00
			Maximum			121.00		12.10		234.00		30.22		93.00

BMI, body mass index; CAP, controlled attenuation parameter; CRP, C-reactive protein; GFR, glomerular filtration rate; HDL, high-density lipoprotein; LDL, low-density lipoprotein; LSM by VCTE, liver stiffness measurement by vibration-controlled transient elastography; TG, triglycerides.

**Table 4:** Correlations with age.

Correlations		
		Age
Age	Pearson correlation	1
Total cholesterol mg/dL	Pearson correlation	0.007
	Significance: twosided	0.953
	N	77
LDL mg/dL	Pearson correlation	0.125
	Significance: twosided	0.542
	N	26
HDL mg/dL	Pearson correlation	−0.062
	Significance: twosided	0.595
	N	75
TG mg/dL	Pearson correlation	0.123
	Significance: twosided	0.299
	N	73
Glucose mg/dL	Pearson correlation	0.313
	Significance: twosided	<0.001
	N	217
BMI kg/m^2^	Pearson correlation	0.188
	Significance: twosided	0.006
	N	211
GFR mL/min./1.73 m^2^	Pearson correlation	−0.149
	Significance: twosided	0.028
	N	216
CRP mg/L	Pearson correlation	−0.153
	Significance: twosided	0.083
	N	130
LSM by VCTE kPa	Pearson correlation	0.228
	Significance: twosided	<0.001
CAP dB/m	Pearson correlation	0.211
	Significance: twosided	0.025

BMI, body mass index; CAP, controlled attenuation parameter; CRP, C-reactive protein; GFR, glomerular filtration rate; HDL, high-density lipoprotein; LDL, low-density lipoprotein; LSM by VCTE, liver stiffness measurement by vibration-controlled transient elastography; TG, triglycerides.

**Table 5:** Analysis of the prevalence of cardiovascular disease, hypertension, diabetes and smoking in the study group (N = 217). (1) Analysis of the prevalence of cardiovascular disease, hypertension, diabetes and smoking in the study group by age. (2) Analysis of the prevalence of cardiovascular disease, hypertension, diabetes and smoking in the study group by age and gender.

	Cigarette Smoking, N (%)	Diabetes, N (%)		Chronic Coronary Syndrome /Heart Attack/Coronary Interventions-PTCA, CABG/Brain Stroke/TIA, N (%)	Hypertension, N (%)
**No**	171 (79.3%)	205 (94.5%)		217 (100%)	191 (88.0%)
**Yes**	45 (20.7%)	12 (5.5%)		0 (0%)	26 (12.0%)
**(1)**					
**Age in Ranges**	**Condition**	**Cigarette Smoking,** **N (%)**		**Diabetes, N (%)**	**Hypertension,** **N (%)**
Age 20 to 30	No	16 (72.7%)		22 (100.0%)	19 (86.4%)
	Yes	6 (27.3%)		0 (0.0%)	3 (13.6%)
	Total	22 (100.0%)		22 (100.0%)	22 (100.0%)
Age 30 to 40	No	112 (83.0%)		130 (96.3%)	124 (91.9%)
	Yes	23 (17.0%)		5 (3.7%)	11 (8.1%)
	Total	135 (100.0%)		135 (100.0%)	135 (100.0%)
Age 40 to 45	No	44 (73.3%)		53 (88.3%)	48 (80.0%)
	Yes	16 (26.7%)		7 (11.7%)	12 (20.0%)
	Total	60 (100.0%)		60 (100.0%)	60 (100.0%)
**(2)**					
**Gender**	**Age in Ranges**	**Condition**	**Cigarette Smoking,** **N (%)**	**Diabetes, N (%)**	**Hypertension,** **N (%)**
Women	Age 20 to 30	No	8 (80%)	10 (100%)	10 (100%)
		Yes	2 (20%)	0 (0%)	0 (0%)
		Total	10 (100%)	10 (100%)	10 (100%)
Women	Age 30 to 40	No	56 (86.2%)	65 (100%)	62 (95.4%)
		Yes	9 (13.8%)	0 (0%)	3 (4.6%)
		Total	65 (100%)	65 (100%)	65 (100%)
Women	Age 40 to 45	No	17 (81%)	19 (95.5%)	15 (71.4%)
		Yes	4 (19%)	2 (9.5%)	6 (28.6%)
		Total	21 (100%)	21 (100%)	21 (100%)
Men	Age 20 to 30	No	8 (66%)	12 (100%)	9 (75%)
		Yes	4 (33.3%)	0 (0%)	3 (25%)
		Total	12 (100%)	12 (100%)	12 (100%)
Men	Age 30 to 40	No	55 (78.6%)	65 (92,9%)	62 (88.6%)
		Yes	14 (20%)	5 (7.1%)	8 (11.4%)
		Total	69 (98.6%)	70 (100%)	70 (100%)
Men	Age 40 to 45	No	27 (69.2%)	34 (87.2%)	33 (84.6%)
		Yes	12 (30.8%)	5 (12.8%)	6 (15.4%)
		Total	39 (100%)	39 (100%)	39 (100%)

## Data Availability

Data are contained within the article.
